# The Apoptogenic Toxin AIP56 Is a Metalloprotease A-B Toxin that Cleaves NF-κb P65

**DOI:** 10.1371/journal.ppat.1003128

**Published:** 2013-02-28

**Authors:** Daniela S. Silva, Liliana M. G. Pereira, Ana R. Moreira, Frederico Ferreira-da-Silva, Rui M. Brito, Tiago Q. Faria, Irene Zornetta, Cesare Montecucco, Pedro Oliveira, Jorge E. Azevedo, Pedro J. B. Pereira, Sandra Macedo-Ribeiro, Ana do Vale, Nuno M. S. dos Santos

**Affiliations:** 1 Fish Immunology and Vaccinology, Instituto de Biologia Molecular e Celular (IBMC), Universidade do Porto, Porto, Portugal; 2 Instituto de Ciências Biomédicas Abel Salazar (ICBAS), Universidade do Porto, Porto, Portugal; 3 Protein Production and Purification, Instituto de Biologia Molecular e Celular (IBMC), Universidade do Porto, Porto, Portugal; 4 Center for Neuroscience and Cell Biology (CNC), University of Coimbra, Coimbra, Portugal; 5 Chemistry Department, Faculty of Science and Technology, University of Coimbra, Coimbra, Portugal; 6 Dipartimento di Scienze Biomediche dell'Università di Padova and Instituto di Neuroscienze del CNR, Padova, Italy; 7 Organelle Biogenesis and Function, Instituto de Biologia Molecular e Celular (IBMC), Universidade do Porto, Porto, Portugal; 8 Biomolecular Structure, Instituto de Biologia Molecular e Celular (IBMC), Universidade do Porto, Porto, Portugal; 9 Protein Crystallography, Instituto de Biologia Molecular e Celular (IBMC), Universidade do Porto, Porto, Portugal; University of California Los Angeles, United States of America

## Abstract

AIP56 (apoptosis-inducing protein of 56 kDa) is a major virulence factor of *Photobacterium damselae piscicida* (*Phdp*), a Gram-negative pathogen that causes septicemic infections, which are among the most threatening diseases in mariculture. The toxin triggers apoptosis of host macrophages and neutrophils through a process that, *in vivo*, culminates with secondary necrosis of the apoptotic cells contributing to the necrotic lesions observed in the diseased animals. Here, we show that AIP56 is a NF-κB p65-cleaving zinc-metalloprotease whose catalytic activity is required for the apoptogenic effect. Most of the bacterial effectors known to target NF-κB are type III secreted effectors. In contrast, we demonstrate that AIP56 is an A-B toxin capable of acting at distance, without requiring contact of the bacteria with the target cell. We also show that the N-terminal domain cleaves NF-κB at the Cys^39^-Glu^40^ peptide bond and that the C-terminal domain is involved in binding and internalization into the cytosol.

## Author Summary

The apoptosis inducing protein of 56 kDa (AIP56) is a key virulence factor secreted by *Photobacterium damselae piscicida* (*Phdp*), a Gram-negative bacterium that causes septicaemic infections in economically important marine fish species. It is known that AIP56 induces massive destruction of the phagocytic cells of the infected host, allowing the extracellular multiplication of the bacteria and contributing to the genesis of the pathology. Here we show that AIP56 acts by cleaving NF-κB p65. The NF-κB family of transcription factors is evolutionarily conserved and plays a central role in the host responses to microbial pathogen invasion, regulating the expression of inflammatory and anti-apoptotic genes. Pathogenic bacteria have evolved complex strategies to interfere with NF-κB signalling, usually by injecting protein effectors directly into the cell's cytosol through bacterial secretion machineries that require contact with host cells. In contrast, AIP56 acts at distance and has an intrinsic ability to reach the cytosol due to the presence of a C-terminal domain that functions as “delivery module.”

## Introduction

The NF-κB family of transcription factors is evolutionarily conserved and comprises five members (NF-κB 1 (p50), NF-κB 2 (p52), RelA (p65), RelB and cRel), which form different combinations of homo- or hetero-dimers [1]. Under normal physiological conditions, NF-κB complexes remain inactive in the cytosol through association with the IκB proteins that mask the nuclear localization domains on NF-κB subunits. A variety of stimuli, including bacterial and viral products and cytokines, acting via cellular receptors such as Toll-like receptors (TLRs), Interleukin-1 receptor (IL-1R) and TNF receptors (TNFRs), trigger a signalling cascade that leads to phosphorylation and degradation of the inhibitory IκB proteins with rapid activation and transport of the NF-κB complexes to the nucleus, resulting in the up-regulation of inflammatory and anti-apoptotic genes [2].

NF-κB activation is considered to be the central initiating event of host responses to microbial pathogen invasion [2]. Therefore, it is not surprising that successful microbial pathogens have evolved complex strategies to interfere with NF-κB signalling. A number of pathogenic bacteria were recently found to interfere with this pathway by targeting different intermediates of the NF-κB activation cascade [2]–[4].

*Photobacterium damselae piscicida* (*Phdp*) is a Gram-negative bacterium that infects several warm water fish species worldwide and is recognized as one of the most threatening pathogens in mariculture [5]–[8]. In acute infections, a rapid septicemia develops and causes very high mortality [5], [6], [9]. Early descriptions of the histopathology of *Phdp* infection recognized the occurrence of cytotoxic alterations [5], [10]–[16] that we found to result from pathogen-induced macrophage and neutrophil apoptotic death [17], [18] by a process that uses mechanisms of the intrinsic and extrinsic apoptotic pathways [19]. The phagocyte destruction observed in *Phdp* infections occurs systemically and culminates in a secondary necrotic process with lysis of the apoptosing cells [17], [18], [20]. This leads to the impairment of host immune defences and to the release of the cytotoxic contents of the phagocytes, contributing to the formation of the necrotic lesions observed in the diseased animals.

We have previously shown that phagocyte apoptosis observed in *Phdp* infections results from the activity of AIP56, a plasmid-encoded exotoxin secreted by virulent strains, and that passive immunization with anti-AIP56 rabbit serum protects against *Phdp* infection [17], [21]. These results implicated AIP56 as a key virulence factor of *Phdp*. However, the molecular target(s) of the toxin remained unidentified and nothing was known about its structure-function relationship.

AIP56 is synthesized as a precursor protein with a cleavable N-terminal signal peptide that is removed during secretion, originating a 497-amino acid mature toxin with the conserved HEIVH zinc-binding motif within its N-terminal region [21], similarly to tetanus neurotoxin [22]. The N-terminal region of AIP56 is homologous to NleC [21], [23], a type III secreted effector present in several enteric pathogenic bacteria, while the C-terminal region is highly similar to an uncharacterized hypothetical protein of *Acrythosiphon pisum* bacteriophage APSE-2 [24] and to the C-terminal portion of a hypothetical protein of the monarch butterfly *Danaus plexippus* (Figure S1). This suggested that AIP56 is a two domain protein, belonging to the group of A-B type toxins that includes diphtheria and tetanus toxins [23], [25], [26].

Recently, it was shown that NleC inhibits NF-κB activation and represses NF-κB-dependent transcription by cleaving NF-κB p65 within its N-terminal region [27]–[31].

Here, we show that AIP56 is a zinc-metalloprotease that cleaves NF-κB p65 and that its enzymatic and apoptogenic activities are correlated. In contrast to NleC, which is delivered into the host cell's cytosol through a type III secretion system, AIP56 is an A-B-type exotoxin with an N-terminal domain responsible for the proteolytic activity and a C-terminal domain involved in binding and internalisation into target cells.

## Results

### The metalloprotease signature of AIP56 is essential for its apoptogenic activity

**Figure 1:**
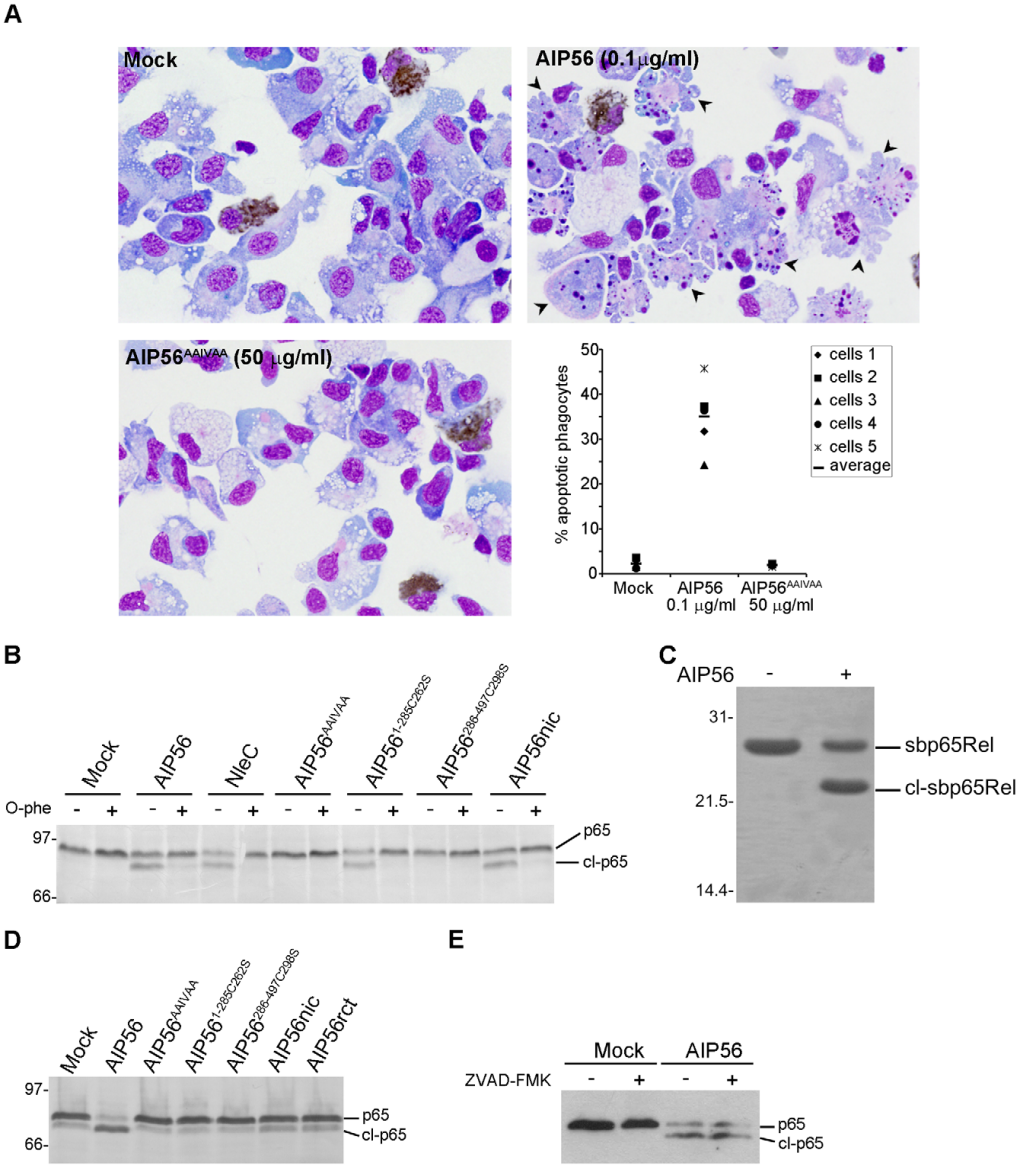
AIP56 is a zinc-metalloprotease that cleaves NF-κB p65 at the Cys^39^-Glu^40^ peptide bond. (**A**) Disruption of the zinc-metalloprotease signature abolishes AIP56 apoptogenic activity. Sea bass peritoneal leukocytes collected from 5 animals were incubated with AIP56 or AIP56^AAIVAA^, for 4 h at 22°C. Mock-treated cells were used as controls. Images shown are representative cytospin preparations stained with Antonow's for labelling neutrophils (brown) followed by Hemacolor. Note the presence of several apoptotic cells (arrowheads) in the sample incubated with AIP56 and their absence in cells incubated with AIP56^AAIVAA^ and in mock-treated cells. The percentage of apoptotic phagocytes, determined by morphological analysis, is depicted in the dot plot. (**B**) Incubation of cell lysates with AIP56, AIP56^1–285C262S^ and nicked AIP56 resulted in p65 cleavage. Lysates were incubated for 2 h at 22°C with 1 µM of the indicated proteins in the presence or absence of the metalloprotease inhibitor 1,10-phenanthroline (O-phe) and p65 cleavage assessed by Western blotting. (**C**) AIP56 cleaves NF-κB p65 at the Cys^39^-Glu^40^ peptide bond. Recombinant sea bass p65Rel (7.5 µM) was incubated in the presence or absence of 1 µM of AIP56 for 3 h at 22°C and analysed by SDS-PAGE. Edman degradation of the cleaved sbp65Rel (cl-sbp65Rel) identified the sequence E^40^GRSA^44^ showing that cleavage occurred after the conserved C^39^. (**D**) Incubation of leukocytes with AIP56, but not with AIP56^AAIVAA^, AIP56^1–285C262S^, AIP56^286–497C298S^, nicked AIP56 (AIP56nic) or reconstituted AIP56 (AIP56rct) leads to p65 depletion. Leukocytes were incubated with 10 µg/ml of the indicated proteins for 2 h at 22°C and p65 cleavage was assessed by Western blotting. (**E**) AIP56-mediated p65 cleavage is caspase-independent. Leukocytes were incubated with 2 µg/ml AIP56 in the presence or absence of the pan-caspase inhibitor Z-VAD-FMK for 2 h at 22°C, and p65 cleavage was assessed by Western blotting. Numbers to the left of the panels refer to the position and mass of the molecular weight markers, in kDa.

In order to clarify the role played by the zinc metalloprotease activity of AIP56, a mutant (AIP56^AAIVAA^) containing a disrupted putative zinc-binding motif was produced. The oligomerization state and secondary structure content of the toxin were undisturbed by the introduced mutations (Figure S2) and atomic absorption spectroscopy did not detect zinc in AIP56^AAIVAA^, while in AIP56 equimolar amounts of zinc (0.93±0.04 mol zinc/mol protein) were present. When tested *ex vivo*, AIP56^AAIVAA^ failed to induce apoptosis of sea bass phagocytes, whilst a large number of cells with apoptotic morphology were observed after treatment with AIP56 (Figure 1A). These results indicate that an intact metalloprotease domain is essential for the apoptogenic activity of AIP56.

It is worth noting that the AIP56 concentrations used in the present work are biologically relevant, since they are similar to those detected in the plasma of infected fish (Figure S3).

### AIP56 is a zinc dependent metalloprotease that cleaves NF-κB p65 at the Cys^39^-Glu^40^ peptide bond

When incubated with sea bass cell lysates, AIP56 cleaved p65 with the appearance of a lower MW fragment (Figure 1B). Proteolysis of p65 did not occur in cell lysates incubated with AIP56^AAIVAA^ or with AIP56 in the presence of the metalloprotease inhibitor 1,10-phenanthroline (Figure 1B). The p65 fragment was recognised by an antibody specific for a peptide located at the C-terminal region of p65 indicating that the AIP56-mediated p65 cleavage occurred within the N-terminal region, where the Rel-homology domain is located. To map the cleavage site, recombinant sea bass p65Rel domain (sbp65Rel) was incubated with the toxin. SDS-PAGE analysis showed that AIP56 cleaved recombinant sbp65Rel *in vitro* (Figure 1C), and N-terminal sequencing of the cleaved fragment revealed that the cleavage occurred at the Cys^39^-Glu^40^ peptide bond, similar to what was described for NleC [27]. Experiments using *in vitro* synthesised ^35^S-labeled sea bass p65Rel domain (sbp65Rel) and three sbp65Rel mutants (sbp65RelC39A, sbp65RelE40A and sbp65CE39-40AA) showed that mutation of the evolutionarily conserved Cys^39^ had no effect on p65 cleavage by either AIP56 or NleC (Figure S4). However, mutation of the following Glu^40^ inhibited cleavage and double mutation of Cys^39^ and Glu^40^ completely abolished p65 proteolysis by AIP56 and NleC (Figure S4).

To determine if cellular intoxication by AIP56 involves cleavage of NF-κB p65, sea bass peritoneal leukocytes were incubated with wild type toxin or with AIP56^AAIVAA^ mutant and p65 proteolysis assessed by Western blotting. Wild type AIP56 caused NF-κB p65 depletion, whilst AIP56^AAIVAA^ was inactive (Figure 1D). It has been reported that caspase-3 can cleave p65 [32], [33]. To investigate whether caspases are involved in AIP56-dependent cleavage of p65, cells were incubated with the toxin in the presence or absence of the pan-caspase inhibitor ZVAD-FMK (Figure 1E), previously shown to block AIP56-induced apoptosis [19]. In these experiments, ZVAD-FMK was effective in protecting cells from AIP56-induced apoptosis (data not shown), but did not affect NF-κB p65 cleavage (Figure 1E), indicating that AIP56-mediated p65 depletion is a caspase-independent event. Taken together, the above results demonstrate that the metalloprotease activity of AIP56 is responsible for the cleavage of NF-κB p65 at the Cys^39^-Glu^40^ peptide bond.

### AIP56 has two domains

**Figure 2:**
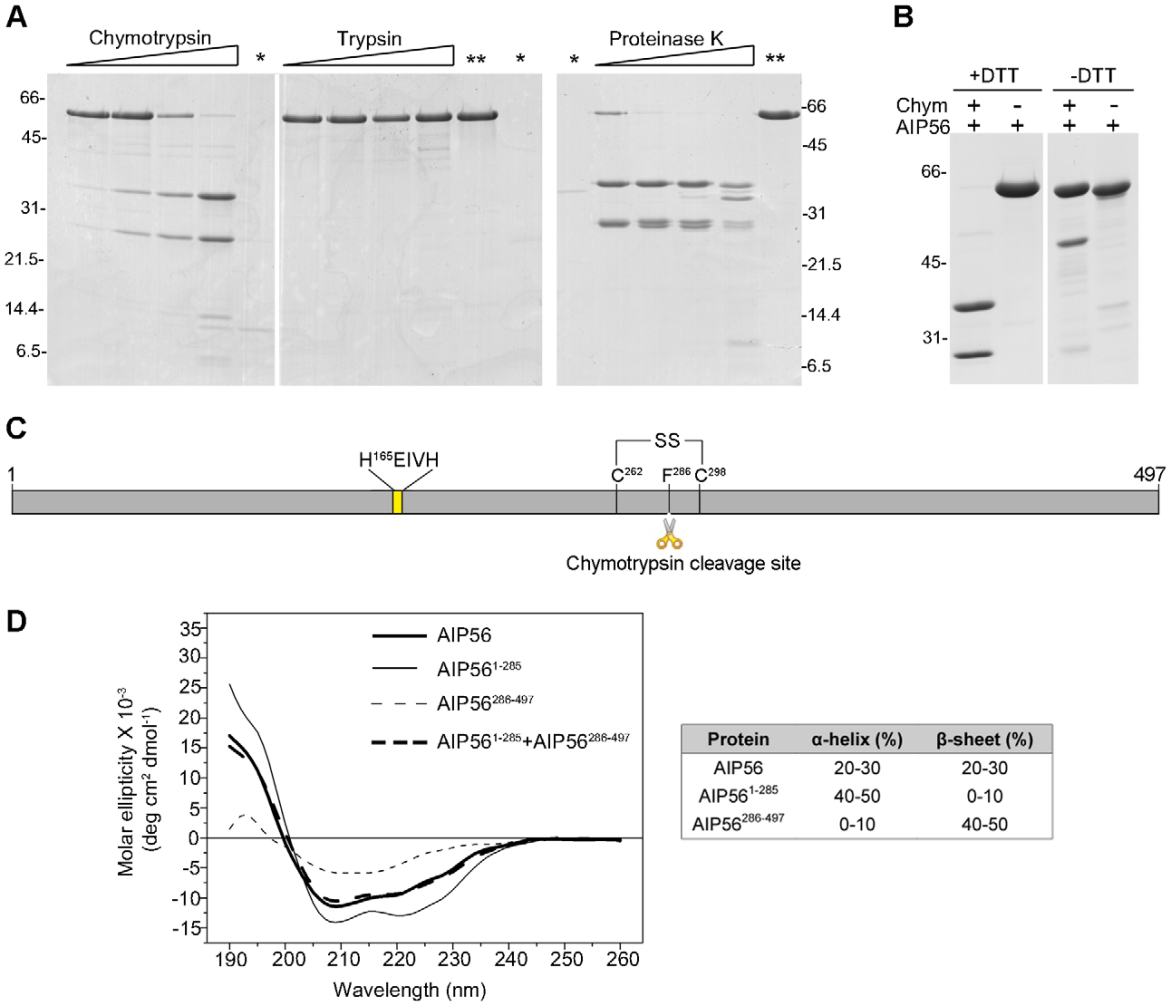
AIP56 is composed of two domains linked by a disulphide bridge. (**A**) Limited proteolysis of AIP56 with chymotrypsin and proteinase K produces two major fragments. AIP56 (0.6 mg/ml) was incubated with 0.25, 1.25, 6.25 or 25 µg/ml of chymotrypsin, trypsin and proteinase K for 30 min on ice and digests analysed by reducing SDS-PAGE. The proteases (marked as *) and undigested AIP56 (marked as **) were loaded as controls. (**B**) The two AIP56 digestion fragments are linked by a disulphide bridge. AIP56 was incubated with or without 25 µg/ml chymotrypsin (Chym) for 30 min on ice and digests analysed under reducing (+DTT) or non-reducing (−DTT) SDS-PAGE. Numbers to the left and right of the panels refer to the position and mass of the molecular weight markers, in kDa. (**C**) Schematic representation of AIP56. (**D**) Far-UV CD spectra of AIP56 (thick solid line), AIP56^1–285^ (thin solid line), AIP56^286–497^ (thin dashed line) and the weighted sum of AIP56^1–285^ and AIP56^286–497^ spectra (thick dashed line).

The primary structure of AIP56 suggests that this toxin comprises two functional domains and could be an A-B toxin with its two moieties linked by a single disulphide bond (Figure S1) [23]. Therefore, in order to define domain boundaries within the toxin, limited proteolysis experiments were performed. SDS-PAGE analysis of AIP56 digested with chymotrypsin, trypsin or proteinase K revealed that the toxin is highly resistant to trypsin digestion, whereas chymotrypsin and proteinase K cleaved AIP56 into two major fragments with approximately 32 and 24 kDa (Figure 2A). These two fragments were only detected upon treatment with the reducing agent DTT, suggesting that they are linked by a disulphide bridge (Figure 2B). N-terminal Edman sequencing revealed that chymotrypsin cleavage occurred between Phe^285^ and Phe^286^, in the amino-acid stretch flanked by the two unique cysteine residues (Cys^262^ and Cys^298^) of AIP56 (Figure 2C). Altogether, these results indicate that AIP56 is composed of two domains linked by a disulphide bridge.

### The N-terminal domain is responsible for the catalytic activity and the C-terminal domain is implicated in binding to target cells

To better understand the function of the two AIP56 domains, constructs corresponding to the N- and C-terminal portions of the toxin (AIP56^1–285^ and AIP56^286–497^, respectively) were designed, taking into account the boundary defined by the chymotrypsin cleavage site (Figure 2C). Purification of these two recombinant proteins using the experimental conditions used for the full-length toxin revealed that they display a major propensity to oxidize leading to the formation of DTT-sensitive dimers (Figure S5A), a phenomenon that could have a functional impact and complicate subsequent analyses. Therefore, versions of the constructs with the single cysteine replaced by serines (AIP56^1–285C262S^ and AIP56^286–497C298S^, respectively) were produced. The mutants are undistinguishable from the non-mutant proteins, as assessed by CD (Figure S5B and S5C) with the N-terminal domain composed mainly of α-helices, whereas β–sheet is the predominant secondary structure of the C-terminal moiety (Figure 2D). Furthermore, the weighted sum of the CD spectra of the N- and C-terminal domains reproduces the spectrum of the entire protein (Figure 2D), indicating conservation of the native structure.

**Figure 3:**
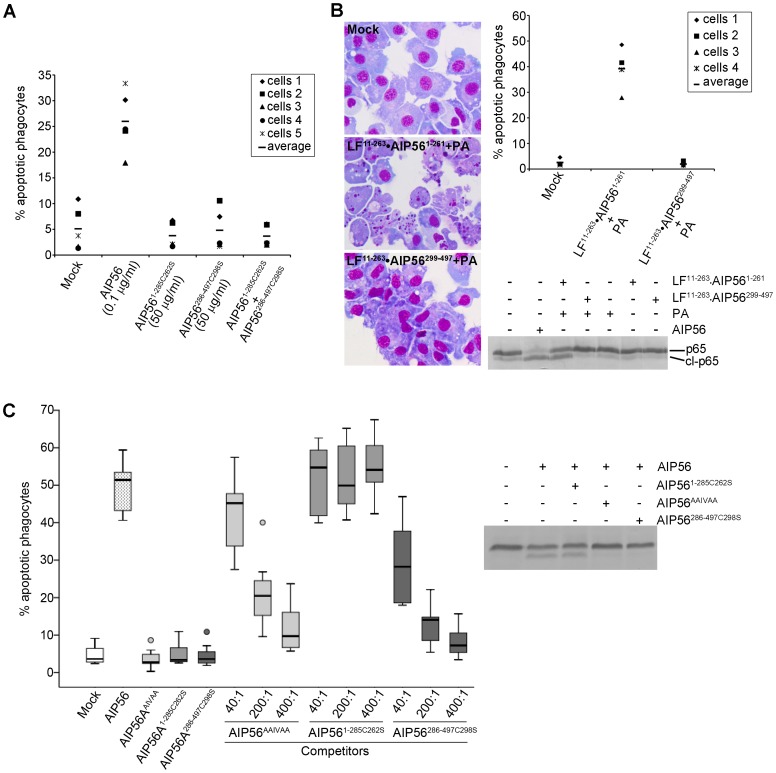
AIP56 N-terminal domain plays the catalytic role and the C-terminal domain is involved in binding and entry into cells. (**A**) AIP56^1–285C262S^ and AIP56^286–497C298S^ lack apoptogenic activity. Leukocytes collected from 5 animals were incubated with AIP56, AIP56^1–285C262S^, AIP56^286–497C298S^, or a mixture of AIP56^1–285C262S^ and AIP56^286–497C298S^ (50 µg/ml each) for 4 h at 22°C. The percentage of apoptotic phagocytes was determined by morphological analysis of cytospin preparations stained with Hemacolor. (**B**) Delivery of AIP56 N-terminal domain into the cell's cytosol using *B. anthracis* LF/PA system reproduces the activity of full length AIP56. Leukocytes from 4 animals were incubated for 4 h at 22°C with 20 nM LF^11–263^•AIP56^1–261^ or LF^11–263^•AIP56^299–497^ in the presence of 10 nM PA. Cells incubated with 2 µg/ml (35 nM) AIP56 were used as positive control. Cleavage of NF-κB p65 was detected by Western blotting and the occurrence of apoptosis by morphological analysis of cytospin preparations stained with Hemacolor. Note the presence of several apoptotic cells in the samples incubated with PA+LF^11–263^•AIP56^1–261^. (**C**) AIP56 C-terminal domain is involved in toxin binding and entry into the target cells. AIP56^AAIVAA^ and AIP56^286–497C298S^, but not AIP56^1–285C262S^, inhibit AIP56-associated p65 cleavage and apoptogenic activity. Leukocytes collected from 7 fish were incubated with AIP56^AAIVAA^, AIP56^1–285C262S^ or AIP56^286–497C298S^ at final concentrations of 0.35, 1.75 or 3.5 µM for 15 min on ice, followed by further 15 min incubation on ice with 8.75 nM (0.5 µg/ml) AIP56 in the presence of the competitors. The competitor:AIP56 molar ratios are indicated. Cells incubated with AIP56 in the absence of competitors or with 3.5 µM of each competitor alone were used as controls. Cells were washed, transferred to 22°C and incubated for 4 h prior to determination of the percentage of apoptotic cells by morphological analysis of cytospin preparations stained with Hemacolor. Left panel presents the box plot of percentage of apopotic cells (the middle bar corresponds to the median and the lower and upper side of the boxes, the first and third quartiles; circles signal extreme observations). The inhibitory effect of the highest dose of each competitor upon AIP56-mediated cleavage of p65 was assessed by Western blotting.

To test the catalytic activities of the AIP56 N- and C-terminal domains, AIP56^1–285C262S^ and AIP56^286–497C298S^ were incubated with fish leukocyte lysates (Figure 1B) or with *in vitro* translated ^35^S-labeled sbp65Rel (Figure S5D). The C-terminal construct did not display catalytic activity, whereas the N-terminal domain cleaved p65, similarly to the full-length toxin. However, neither changes in cellular p65 levels (Figure 1D) nor apoptosis (Figure 3A) were observed in sea bass leukocytes incubated with the N- or C-terminal truncate or with a mixture of both. This indicates that the two AIP56 domains are non-toxic and suggests that they need to be part of the same molecule to elicit a biological effect.

The cytosolic location of NF-κB p65 could mean that the lack of toxicity of the N-terminal domain was related to its inability to enter the cells and reach its target. Hence, a strategy to deliver the N-terminal domain into the cell cytosol was designed. Chimeric proteins consisting of the N-terminal portion of *Bacillus anthracis* LF fused to the AIP56 protease domain (LF^11–263^•AIP56^1–261^) or to the C-terminal domain (LF^11–263^•AIP56^299–497^) were produced. Intoxication assays were performed in the presence of PA, the receptor-binding subunit for LF [34]. In cells incubated with LF^11–263^•AIP56^1–261^ the p65 levels were significantly reduced, confirming that LF^11–263^•AIP56^1–261^ was successfully delivered into the cell cytosol, while no changes in p65 levels were observed in cells incubated with LF^11–263^•AIP56^299–497^ (Figure 3B). Accordingly, LF^11–263^•AIP56^299–497^ did not display apoptogenic activity, while incubation with LF^11–263^•AIP56^1–261^ resulted in an increased number of cells with apoptotic morphology (Figure 3B), similar to what was observed in cells incubated with AIP56. Thus, delivery of the AIP56 N-terminal domain into the cytosol reproduces the toxic effect of the full length toxin, confirming that this domain is responsible for the toxin's catalytic and apoptogenic activities. These results also suggest that the C-terminal domain of AIP56 is involved/required for entrance of the toxin into cells. To investigate this possibility, AIP56^AAIVAA^, AIP56^1–285C262S^ or AIP56^286–497C298S^ were used in competition experiments with AIP56. Both p65 cleavage and apoptosis were monitored in these experiments. AIP56^286–497C298S^ and AIP56^AAIVAA^, but not AIP56^1–285C262S^, were able to inhibit the apoptogenic activity of wild type AIP56 in a dose-dependent manner (Figure 3C, left panel). Furthermore, AIP56^AAIVAA^ and AIP56^286–497C298S^ inhibited AIP56-mediated p65 degradation, whereas no effect could be observed when AIP56^1–285C262S^ was used as competitor (Figure 3C, right panel). These results indicate that the C-terminal domain mediates binding of the toxin to the cell surface and entry into the cells.

### Translocation of AIP56 requires integrity of the Cys^262^-Cys^298^ linker but the disulphide bridge is not an absolute requirement for toxicity

**Figure 4:**
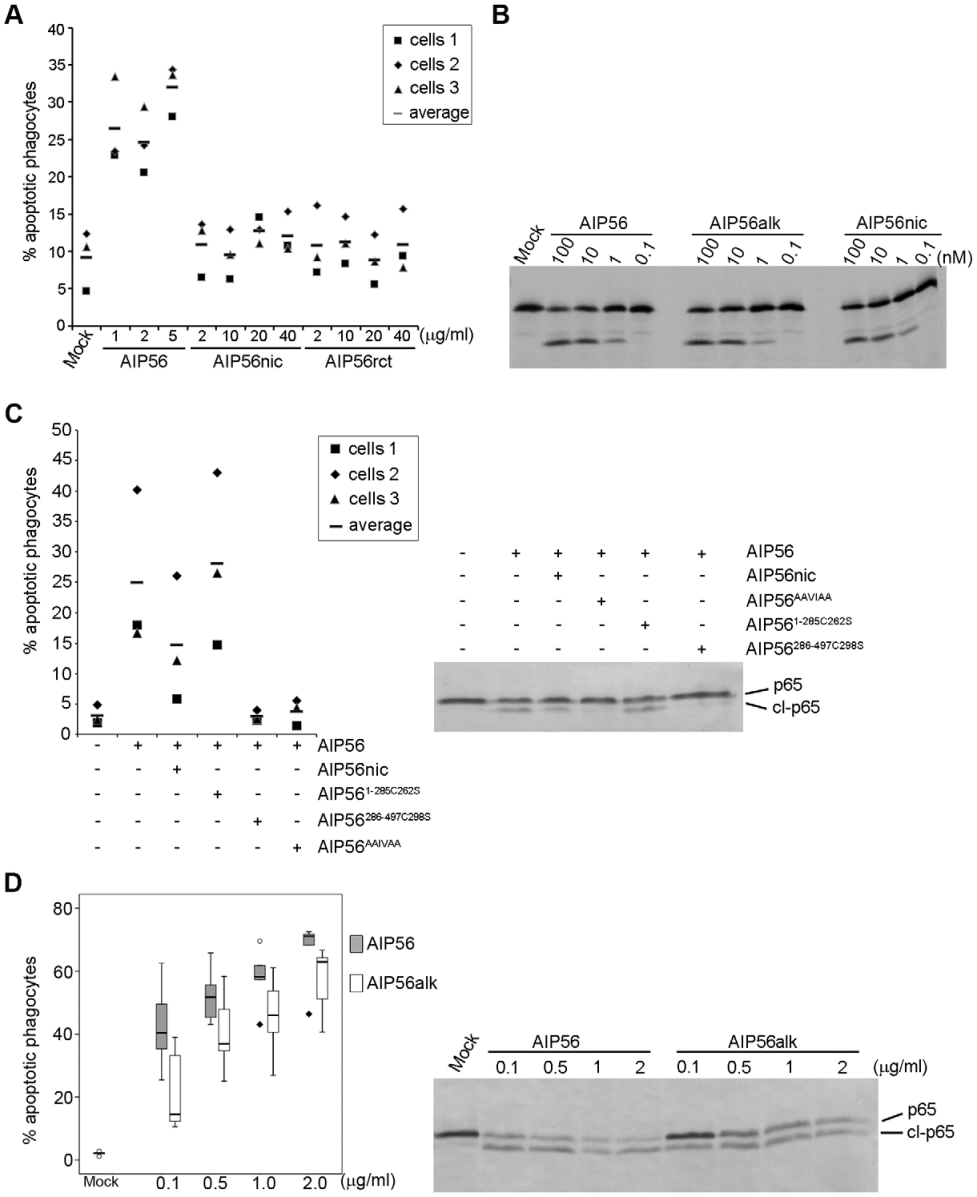
AIP56 toxicity requires integrity of the linker but the disulfide bridge is dispensable for intoxication. (**A**) Nicked and reconstituted AIP56 are not apoptogenic. Leukocytes collected from 3 animals were incubated with nicked or reconstituted AIP56 (AIP56nic and AIP56rct, respectively) for 4 h at 22°C and the percentage of apoptotic cells determined by morphological analysis of cytospin preparations stained with Hemacolor. Cells treated with AIP56 and mock-treated cells were used as positive and negative controls, respectively. (**B**) Nicked and alkylated AIP56 (AIP56alk) display proteolytic activity *in vitro* in the same dose range as AIP56. ^35^S-labeled sbp65Rel was incubated for 2 h at 22°C with wild type, nicked or alkylated AIP56 and cleavage assessed by autoradiography. (**C**) Nicked AIP56 competes with intact AIP56 and inhibits its toxicity. Leukocytes collected from 3 animals were incubated with 3.5 µM nicked AIP56 (AIP56nic) for 15 minutes on ice followed by further 15 min incubation on ice with 8.75 nM of AIP56. Mock-treated cells, cells incubated with 8.75 nM AIP56, or cells incubated with 3.5 µM of AIP56^AAIVAA^, AIP56^1–285^ or AIP56^286–497^ before incubation with 8.75 nM of AIP56 were used as controls. Cells were washed, transferred to 22°C and incubated for 4 h. The percentage of apoptotic cells was determined by morphological analysis of cytospin preparations stained with Hemacolor and the p65 cleavage was assessed by Western blotting. (**D**) Disruption of the disulphide bridge linking Cys^262^ and Cys^298^ partially compromises AIP56 toxicity. Leukocytes collected from 5 animals were incubated with AIP56 or AIP56alk for 4 h at 22°C and the percentage of apoptotic cells determined by morphological analysis of cytospin preparations stained with Hemacolor. Left panel presents the box plot of percentage of apopotic cells (the middle bar corresponds to the median and the lower and upper side of the boxes, the first and third quartiles; circles and diamonds signal extreme observations). When used at the same concentration, AIP56alk resulted in lower percentage of apoptotic cells than AIP56, except for the dose of 0.5 µg/ml, where no statistical differences were observed.

Results obtained in experiments using N- and C-terminal truncates of AIP56 suggested that the two domains must be part of the same molecule to display toxicity. In order to investigate if the two domains of the toxin bound by a disulphide bridge are able to intoxicate cells, we nicked the toxin with chymotrypsin. Nicking of the toxin and integrity of the disulphide bridge linking the two fragments were confirmed by reducing and non-reducing SDS-PAGE (Figure S6A). Surprisingly, no changes in p65 cellular levels (Figure 1D) and no apoptosis (Figure 4A) were observed upon incubation of sea bass peritoneal cells with nicked toxin. Similar results were obtained using a reconstituted version of the toxin (Figure 1D and 4A) consisting of disulphide-bound AIP56^1–285^/AIP56^286–497^ along with trace amounts of AIP56^1–285^ and of AIP56^286–497^ homodimers and monomers (Figure S6A). Although nicking abolished cellular toxicity, it did not induce major structural changes (Figure S6B) and only a 1°C decrease in Tm (39±0.13°C for AIP56 and 38±0.25°C for nicked AIP56; mean±SD of 16 measurements in four independent experiments) was measured by DSF. More importantly, nicked AIP56 retained both proteolytic activity against p65 *in vitro* (Figure 1B and 4B) and cell binding ability, as indicated by the partial inhibition of the AIP56-mediated p65 cleavage and apoptosis in competition experiments (Figure 4C). These results suggest that the integrity of the linker region between the two cysteine residues is needed for toxin internalization, in contrast to what is known for the diphtheria, tetanus and botulinum toxins, where nicking of the inter-cysteine loop is required for toxicity [26], [35].

In tetanus and botulinum neurotoxin type A, it has been shown that the disulphide bridge is essential for neurotoxicity [36], [37]. We found that disruption of the disulphide bridge linking Cys^262^ and Cys^298^ of AIP56 by alkylation (Figure S6D) did not affect the catalytic activity of the toxin *in vitro* (Figure 4B), but partially compromised its toxicity (Figure 4D), suggesting that in AIP56 the disulphide bridge plays a role in the intoxication process but is not an absolute requirement for toxicity.

## Discussion

In this study, we report the structural and functional characterization of AIP56 as an A-B type bacterial exotoxin that cleaves NF-κB p65. Considering the anti-apoptotic functions of NF-κB, and in particular, of its p65 subunit [38]–[40], AIP56-mediated depletion of NF-κB p65 likely explains the disseminated phagocyte apoptosis observed in *Phdp* infections that contributes to subvert the host immune response and determines the outcome of the infection [17], [19], [21]. This adds to a general theme of host-pathogen interaction that has recently emerged, consisting in the induction of apoptosis of the host immune cells to the pathogen advantage [41]–[43].

Here we demonstrate that, similar to the anthrax lethal factor and to the clostridial neurotoxins [44], [45], AIP56 is a zinc-endopeptidase but with a catalytic activity towards NF-κB p65. Furthermore, we show that AIP56 is organized into two distinct domains linked by a single disulphide bond. The N-terminal domain of AIP56 harbours the catalytic activity of the toxin and cleaves NF-κB p65 at the Cys^39^-Glu^40^ peptide bond, within the p65 N-terminal Rel homology domain, where several key residues of p65 known to be involved in DNA interaction are located [46]–[48]. In the last decade, several reports revealed that Cys^38^ of human p65 (Cys^39^ in sea bass p65) interacts with the phosphate backbone of NF-κB binding sites [46], that its oxidation and nitrosylation inhibit DNA binding [49] and that it is targeted by several inhibitors of NF-κB with anti-inflammatory and/or anticancer properties [50]–[60]. More recently, it was shown that hydrogen sulphide-linked sulfhydration of Cys^38^ of human p65 plays a key role in regulating the anti-apoptotic actions of NF-κB [40]. Therefore, cleavage of sea bass p65 by AIP56 disrupts a segment crucial for DNA interaction. Considering that the proteolytic activity of AIP56 towards p65 is similar to the one previously described for NleC (both proteases cleave p65 at the same peptide bond), and based on the observation that p65 cleavage by NleC compromises NF-κB dependent transcription [27], [28], [30], it is likely that AIP56 also affects NF-κB transcriptional activity.

Although in many cell types down-regulation of NF-κB is not sufficient to trigger apoptosis, it is widely recognised that cells with inactivated NF-κB are more prone to commit suicide in response to different stimuli, including TNF-α and TLRs ligands [38], [61]–[63], and that the anti-apoptotic actions of NF-κB can be largely attributed to its p65 subunit [38]–[40]. In the context of bacterial infections, inhibition of NF-κB function usually leads to impairment of the inflammatory responses [2]. The induction of apoptosis by bacterial effectors through interference with NF-κB activity has also been described, but is a far less common scenario. Examples are *Yersinia* YopP/J [64]–[66] and *Aeromonas salmonicida* Aop [67], both inhibiting the degradation of the inhibitory IκB proteins [64], [68]–[71], and *V. parahaemolyticus* protein VP1686 that interacts with and suppresses DNA binding activity of NF-κB [72]. It remains to be determined whether AIP56-mediated depletion of p65 is sufficient to induce apoptosis, in resemblance to what has been suggested for the macrophage apoptosis induced by *V. parahaemolyticus* type III secreted effector VP1686 [72], or if it requires an additional stimulus.

Almost all bacterial effectors that have been described to target NF-κB signalling are injected directly into the host cell cytosol by type III or type IV secretion systems (see reviews by [2], [4]). In contrast, we have found that the AIP56 N-terminal metalloprotease can only act when linked to a C-terminal binding domain that, by analogy with other A-B toxins, may assist the protease domain in its membrane translocation into the cytosol [34], [73], [74].

Bacterial A-B toxins are often secreted as a single polypeptide chain that is cleaved into the disulphide-bound A and B domains [26], [74]. In these toxins, proteolytic nicking and integrity of the disulphide bond linking the A and B domains are essential for toxicity [19], [35], [36], [75]–[77]. In contrast, AIP56 toxicity is abolished by proteolytic nicking and only mildly compromised by disruption of the disulphide bridge by alkylation. Considering that nicked AIP56 retains the ability to interact with the cell membrane, these observations suggest that the linker region (between Cys^262^ and Cys^298^) is involved in translocating the toxin into the host cell cytosol. The decreased toxicity resulting from alkylation suggests that the integrity of the disulphide bond is important, although not absolutely required, for AIP56 intoxication. The disulphide bond may be involved in stabilizing the spatial relationship between the domains. In addition, that bond is hydrophobic and polarizable and its alkylation can have implications in membrane insertion, as reported for tetanus and botulinum neurotoxins [78].

AIP56 is synthesised as a single polypeptide and, contrary to what has been reported for most A-B toxins, there is no evidence of proteolytically processed toxin in the bacterial culture supernatants or in the serum of infected fish [21]. Furthermore, despite several attempts, we were unable to detect proteolytic processing of AIP56 upon its interaction with host cells. If AIP56 needs to be processed in order to exert its effect, the lack of detection of processed toxin may result from a very small amount of processed toxin (not detectable in our experiments) being sufficient to intoxicate the cells, similarly to what was described for other toxins [79]. Alternatively, after endocytosis, unprocessed AIP56 may be translocated into the cytosol as described for *Pseudomonas* exotoxin A [80], [81] or may localize in an endomembrane (e.g. endosomal membrane) with the catalytic domain facing the cytosolic compartment where it can interact with and cleave p65. Studies aiming at discriminating between these hypotheses will be developed in the future.

It is now recognised that horizontal transfer of entire genes or portion of genes plays a key role in generating diversity in pathogens by allowing them to acquire novel phenotypic characteristics. Indeed, there are several examples of bacterial genes with a mosaic structure, composed of diverse segments with different origins [82]–[87]. The structure of AIP56 suggests that the toxin has a chimeric structure, having an N-terminal catalytic domain highly identical to the type III effector NleC and a C-terminal domain homologous to a hypothetical protein of the bacteriophage APSE-2. The actual transfer events that gave rise to such a chimeric protein toxin remain to be disclosed.

The AIP56 catalytic domain and NleC have the same NF-κB p65 cleavage activity. However, NleC requires a type III secretion machinery for activity, while AIP56 has an intrinsic ability to reach the cytosol, due to the presence of the additional C-terminal domain that functions as a “delivery module”. This difference may have relevant implications when considering the use of both pathogen-derived molecules as therapeutic agents in situations associated with uncontrolled activation of NF-κB such as inflammatory diseases and cancer.

## Materials and Methods

Additional details can be found in Supporting Information.

### Ethics statement

This study was carried out in accordance with European and Portuguese legislation for the use of animals for scientific purposes (Directive 86/609/EEC; Decreto-Lei 129/92; Portaria 1005/92). The work was approved by Direcção Geral de Veterinária, the Portuguese authority for animal protection (ref. 004933, 2011-02-22).

### Fish

Sea bass (*Dicentrarchus labrax*), were kept in a recirculating, ozone-treated salt-water (25–30‰) system at 20±1°C, and fed at a ratio of 2% body weight per day. Fish were euthanized with 2-phenoxyethanol (Panreac; >5 ml/10 L).

### Production and purification of recombinant proteins

DNA coding sequences were cloned into NcoI/XhoI restriction sites of pET-28a(+) (Novagen) as described in Supporting Information. Mutants were generated by site directed mutagenesis using QuickChange Site-Directed Mutagenesis Kit (Stratagene) following manufacturer's instructions. Recombinant His-tagged proteins were expressed in *E. coli* BL21(DE3) cells. AIP56, AIP56^1–285^, AIP56^286–497^, AIP56^286–497C298S^, NleC, LF^11–263^•AIP56^1–261^ and LF^11–263^•AIP56^299–497^ were purified from the soluble fraction of induced bacteria by metal-affinity chromatography. After this step, AIP56, AIP56^1–285^, AIP56^286–497^ were subjected to anion exchange chromatography, whereas AIP56^286–497C298S^ and NleC were subjected to size exclusion chromatography. AIP56^AAIVAA^ and AIP56^1–285C262S^ were purified from inclusion bodies by metal-affinity chromatography under denaturing conditions, refolded by dialysis against sea bass PBS (sbPBS; phosphate buffer saline with osmotic strength adjusted to 322 mOsm) with 10% (v/v) glycerol and purified by size exclusion chromatography. For reconstitution of AIP56, AIP56^1–285^ and AIP56^286–497^ were mixed in equimolar amounts in 8 M urea, 1 mM DTT and refolded by extensive dialysis against sbPBS. Nicked AIP56 was obtained by limited proteolysis with 25 µg/ml chymotrypsin, as described below, followed by metal-affinity chromatography purification. To prepare alkylated toxin, 63 µM AIP56 in 20 mM Tris-HCl pH 8.0, 200 mM NaCl, 10 mM DTT was incubated with 5 mM iodoacetamide (Sigma) for 30 min at RT and dialysed against 20 mM Tris-HCl pH 8.0, 200 mM NaCl.

Sea bass NF-κB p65 REL homology domain (sbp65Rel) was purified from the soluble fraction of induced bacteria by metal-affinity chromatography.

Untagged ^35^S-labeled sbp65Rel and sbp65Rel mutants (sbp65RelC39A, sbp65RelE40A, and sbp65C39E40AA) were produced using the TNT T7 Quick Coupled transcription/Translation kit (Promega), in the presence of Redivue™ L-[35S] methionine (specific activity of 1000 Ci/mmol).

### Limited proteolysis

AIP56 at 0.6 mg/ml in 10 mM Tris-HCl pH 8.0, 200 mM NaCl was incubated with 0.25 to 25 µg/ml trypsin, chymotrypsin or proteinase K (molar ratios of protease:AIP56 of approximately 1∶10 to 1∶1000) for 30 min on ice. Proteases were inactivated by addition of PMSF to a final concentration of 250 µg/ml. Digests were analysed by reducing and non-reducing SDS-PAGE. The two major chymotrypsin digestion fragments were subjected to N-terminal sequencing.

### Circular dichroism spectroscopy (CD)

Far UV CD spectra were acquired on an Olis DSM 20 circular dichroism spectropolarimeter controlled by the Globalworks software. Each spectrum is the average of three scans collected at 20°C with a 0.2 mm path length cuvette and with an integration time of 4 seconds. Proteins were dissolved in 10 mM Tris-HCl, 50 mM NaCl, pH 8.0 and concentrations were determined by absorbance measurements. Analysis of the protein secondary structure was performed using the Globalworks software algorithm.

### Cells

Sea bass peritoneal leukocytes were obtained as previously described [19] and used at a density of 2×10^6^ cells/ml. The peritoneal population of cells consists of approximately 70% macrophages and 20% neutrophils with the presence of small numbers of eosinophilic granular cells, lymphocytes and erythrocytes [19].

### Apoptogenic activity assays

Cells were incubated for 4 h at 22°C with AIP56 or AIP56 derived proteins at the indicated doses. Where indicated, the cells were pre-treated for 30 min at 22°C with 25 µM of the pan-caspase inhibitor N-benzyloxycarbonyl-Val-Ala-Asp(O-Me) fluoromethyl ketone (Z-VAD-FMK). In experiments using LF chimeric proteins, cells were incubated with the indicated concentrations of LF^11–263^•AIP56^1–261^ or LF^11–263^•AIP56^299–497^ with or without 10 nM of anthrax protective antigen (PA) obtained as described [88]. Mock- and AIP56-treated cells were used as controls. Apoptosis was assessed as described [18], by light microscopy morphological analysis of cytospin preparations stained with Hemacolor (Merck) after labelling neutrophils using Antonow's technique [89], [90].

### NF-κB p65 cleavage assays

#### *In vitro*

p65 cleavage was assessed using sea bass peritoneal cell lysates, recombinant or *in vitro* translated ^35^S-labeled sbp65Rel. To prepare cell lysates, cells were incubated in 10 mM Tris-HCl pH 8.0, 150 mM NaCl, 0.5% (v/v) Triton X-100 and 10% (v/v) glycerol for 30 min on ice, briefly sonicated and centrifuged. The supernatant of 2×10^6^ cells was incubated with 1 µM of the indicated proteins for 2 h at 22°C and p65 cleavage evaluated by Western blotting using an anti-sea bass NF-κB p65 rabbit serum (produced using the peptide SIFNSGNPARFVS located at the C-terminal region of sea bass p65 as antigen). Recombinant sbp65Rel (7.5 µM) was incubated for 3 h at 22°C with 1 µM AIP56 and p65 cleavage evaluated by SDS-PAGE. *In vitro* translated ^35^S-labeled sbp65Rel and ^35^S-labeled sbp65Rel mutants (sbp65RelC39A, sbp65RelE40A, and sbp65C39E40AA) were incubated with 0.1, 1, 10 or 100 nM of the different proteins in 10 mM Tris-HCl pH 8.0, 150 mM NaCl and 10% (v/v) glycerol for 2 h at 22°C and p65 cleavage assessed by autoradiography. When specified, 1,10-phenanthroline (Sigma) was used at 5 mM.

#### *Ex vivo*

peritoneal leukocytes were treated for 2 or 4 h as described above in the section “Apoptogenic activity assays”, collected by centrifugation, washed, resuspended in sbPBS and lysed by addition of SDS-PAGE sample buffer. Cleavage of p65 was evaluated by Western blotting, as described above.

### Competition assays

AIP56^AAIVAA^, AIP56^1–285C262S^ and AIP56^286–497C298S^ were tested for their ability to inhibit AIP56's apoptogenic activity and AIP56-mediated p65 cleavage. Cells were pre-incubated for 15 min on ice with different concentrations (350 nM to 3.5 µM) of AIP56^AAIVAA^, AIP56^1–285C262S^, AIP56^286–497C298S^ or nicked AIP56, followed by incubation for further 15 min on ice with 8.75 nM of AIP56 in the presence of the competitors. Unbound proteins were removed by washing with ice cold supplemented L-15 medium [19] and the cells incubated at 22°C for 4 h.

### Statistical analysis

Statistical analysis was performed using a randomized block design, where fish are treated as blocks and the concentration of the treatments/competitors as a factor. The data, percentage of apoptotic cells, have been transformed using the arcsine transformation. Post-hoc comparisons were performed using the Tukey's Honest Significant Difference test. Significance was defined for p<0.05.

## Supporting Information

Figure S1 Schematic diagram of the primary structure of AIP56 and AIP56-related proteins. Grey: signal peptides (experimentally determined for AIP56 [21] and predicted for the remaining proteins using SignalP at http://www.cbs.dtu.dk/services/SignalP/ [91], [92]; Yellow: regions with high identity to NleC and AIP56 N-terminal catalytic domain; Green; regions with high identity to AIP56 linker polypeptide; Orange: regions with high identity to APSE-2 and AIP56 C-terminal domain; Red: zinc-metalloprotease signature HEXXH; White: regions with low identity to AIP56 domains, NleC or APSE-2. Conserved zinc-metalloprotease signature HEXXH, cysteine residues, and other signalled amino acids are represented at their relative positions. AIP56-related proteins were retrieved by Blast analysis of the AIP56 protein sequence against the non-redundant protein sequences database (updated from [23]).
(TIF)

Figure S2 Disruption of the zinc-metalloprotease signature does not induce major structural changes in AIP56. Native-PAGE (**A**) and size exclusion chromatography (**B**) of AIP56 and AIP56^AAIVAA^ showing that disruption of the zinc-binding motif did not affect the monodispersity/stokes radius of the protein. In Native-PAGE, BSA electrophoretic mobility is shown for reference purposes. (**C**) Far-UV CD spectra of wild-type AIP56 (thick line) and AIP56^AAIVAA^ (thin line) showing that the secondary structure content of the toxin was also unaffected by the introduced mutations.
(TIF)

Figure S3 The AIP56 concentration in the plasma of infected fish is in the same range as those used in the present work. (**A**) The presence of AIP56 in plasmas (5 µl aliquots) from sea bass infected with a lethal dose of *Phdp* strain PP3 was determined by Western blotting. Different concentrations of recombinant AIP56 (5 µl) were loaded as standards. Numbers at the left refer to the position and mass (in kDa) of the molecular weight markers. (**B**) Concentrations of AIP56 in the plasmas analysed in (A), determined by densitometry, using a recombinant AIP56 standard curve.
(TIF)

Figure S4 The AIP56 cleavage-determining residues are evolutionarily conserved. (**A**) Mutations in Cys^39^ and Glu^40^ of sbp65Rel inhibit proteolytic processing by AIP56 and NleC. ^35^S-labeled sbp65Rel, sbp65RelC39A, sbp65RelE40A or sbp65CE39-40AA were incubated for 2 h at 22°C with 100 nM of the indicated proteins and cleavage assessed by autoradiography. (**B**) Alignment of the p65 N-terminal region from different species (NCBI accession numbers: *Homo sapiens*, AAA36408; *Mus musculus*, NP_033071; *Gallus gallus*, NP_990460; *Xenopus laevis*, AAH70711; *Monodelphis domestica*, XP_001379658; *Danio rerio*, AAO26404). The residues mutated in (A) are shadowed grey.
(TIF)

Figure S5 Structural and functional analysis of recombinant AIP56 N- and C-terminal domains. (**A**) Reducing and non-reducing SDS-PAGE of purified AIP56^1–285^ and AIP56^286–497^. Numbers at the left refer to the position and mass (in kDa) of the molecular weight markers. (**B**) Far-UV CD spectra of AIP56^1–285^ (thick solid line) and AIP56^1–285C262S^ (thin solid line). (**C**) Far-UV CD spectra of AIP56^286–497^ (thick solid line) and AIP56^286–497C298S^ (thin solid line). (**D**) AIP56, AIP56^1–285C262S^ and NleC cleave sea bass p65 Rel homology domain *in vitro*. ^35^S-labeled sbp65Rel (Met^1^-Arg^188^) was incubated for 2 h at 22°C with 100 nM of the indicated proteins in the presence or absence of the metalloprotease inhibitor 1,10-phenanthroline (O-phe) and cleavage assessed by autoradiography.
(TIF)

Figure S6 Analysis of nicked, reconstituted and alkylated AIP56. (**A**) Reducing (+DTT) and non-reducing (−DTT) SDS-PAGE of AIP56, nicked AIP56 (AIP56nic) and reconstituted AIP56 (AIP56rct). (**B**) Nicking of AIP56 does not affect its secondary structure. Far-UV CD spectra of AIP56 (thick line) and nicked AIP56 (AIP56nic; thin line). (**C**) The culture conditions do not reduce the disulphide bridge of nicked AIP56. Nicked AIP56 was added to a sea bass peritoneal cell suspension in supplemented L-15 medium and incubated up to 4 h at 22°C. An aliquot of nicked toxin not incubated with cells (0) and aliquots of the cell culture supernatant collected 2 or 4 h after incubation with cells (all containing 50 ng of nicked toxin) were run in reducing and non-reducing SDS-PAGE and subjected to Western blotting using an anti-AIP56 rabbit serum. (**D**) Reducing (+DTT) and non-reducing (−DTT) SDS-PAGE of AIP56 and alkylated AIP56 (AIP56alk). Numbers on the left of the panels indicate the mass of the molecular weight markers, in kDa.
(TIF)

Materials and Methods S1 Additional details about the constructs used in this study, protein production and purification, protein quantification, PAGE and Western blotting, analysis of the Zinc content, analytical size exclusion chromatography, circular dichroism spectroscopy (CD), differential scanning fluorimetry (DSF), determination of AIP56 concentration in the plasma of infected fish, are provided as Supporting Materials and Methods.
(DOCX)

Table S1 Primers used in this study.
(DOCX)
