# Supplementary figures and images for: The Apoptogenic Toxin AIP56 Is a Metalloprotease A-B Toxin that Cleaves NF-κb P65
Source: PLoS Pathog. 2013 Feb 28;9(2):e1003128. doi: 10.1371/journal.ppat.1003128 (PMC3585134; doi:10.1371/journal.ppat.1003128)

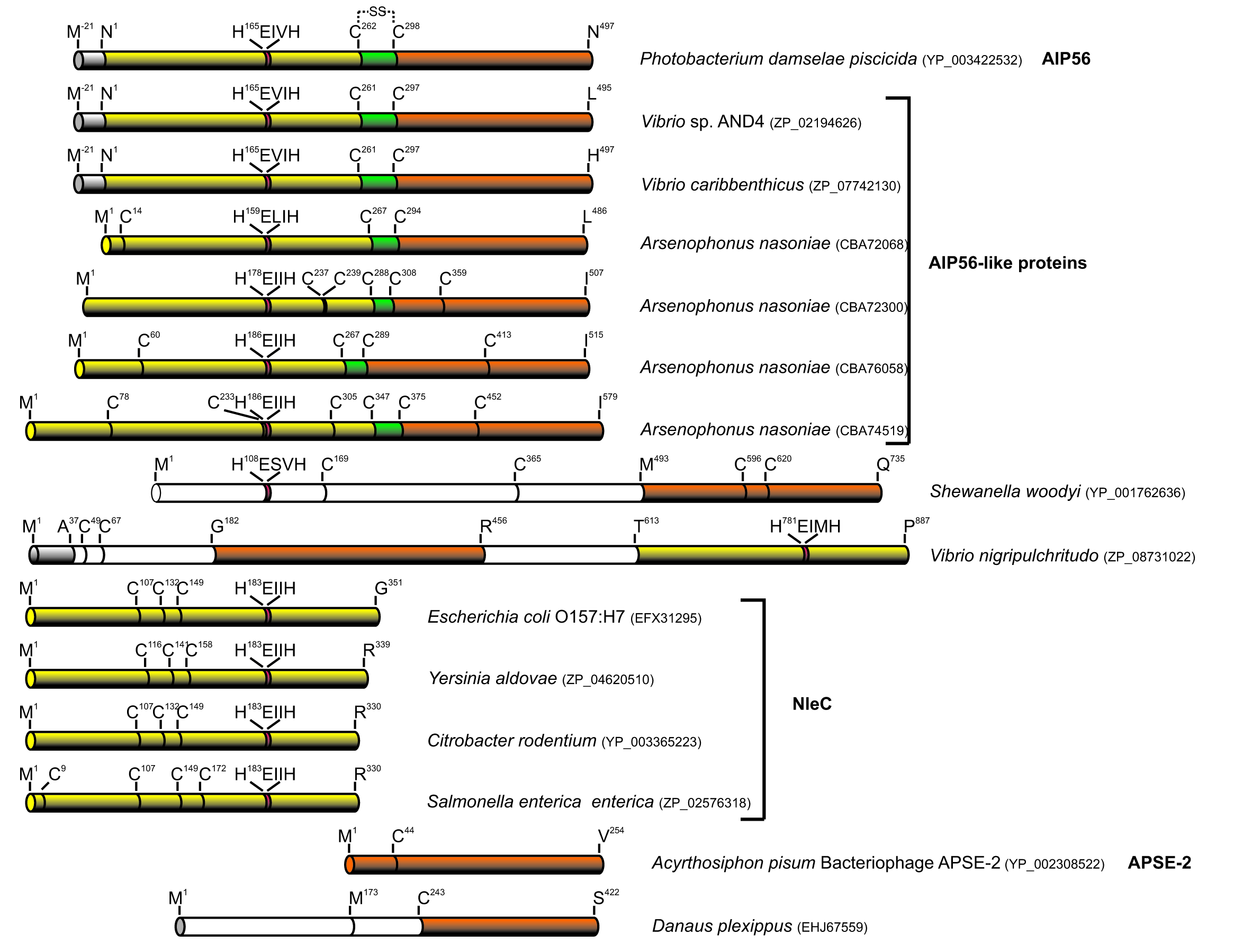

Supplement: Figure S1 — Schematic diagram of the primary structure of AIP56 and AIP56-related proteins. Grey: signal peptides (experimentally determined for AIP56 [21] and predicted for the remaining proteins using SignalP at http://www.cbs.dtu.dk/services/SignalP/ [91], [92]; Yellow: regions with high identity to NleC and AIP56 N-terminal catalytic domain; Green; regions with high identity to AIP56 linker polypeptide; Orange: regions with high identity to APSE-2 and AIP56 C-terminal domain; Red: zinc-metalloprotease signature HEXXH; White: regions with low identity to AIP56 domains, NleC or APSE-2. Conserved zinc-metalloprotease signature HEXXH, cysteine residues, and other signalled amino acids are represented at their relative positions. AIP56-related proteins were retrieved by Blast analysis of the AIP56 protein sequence against the non-redundant protein sequences database (updated from [23]). (TIF) [file ppat.1003128.s001.tif]

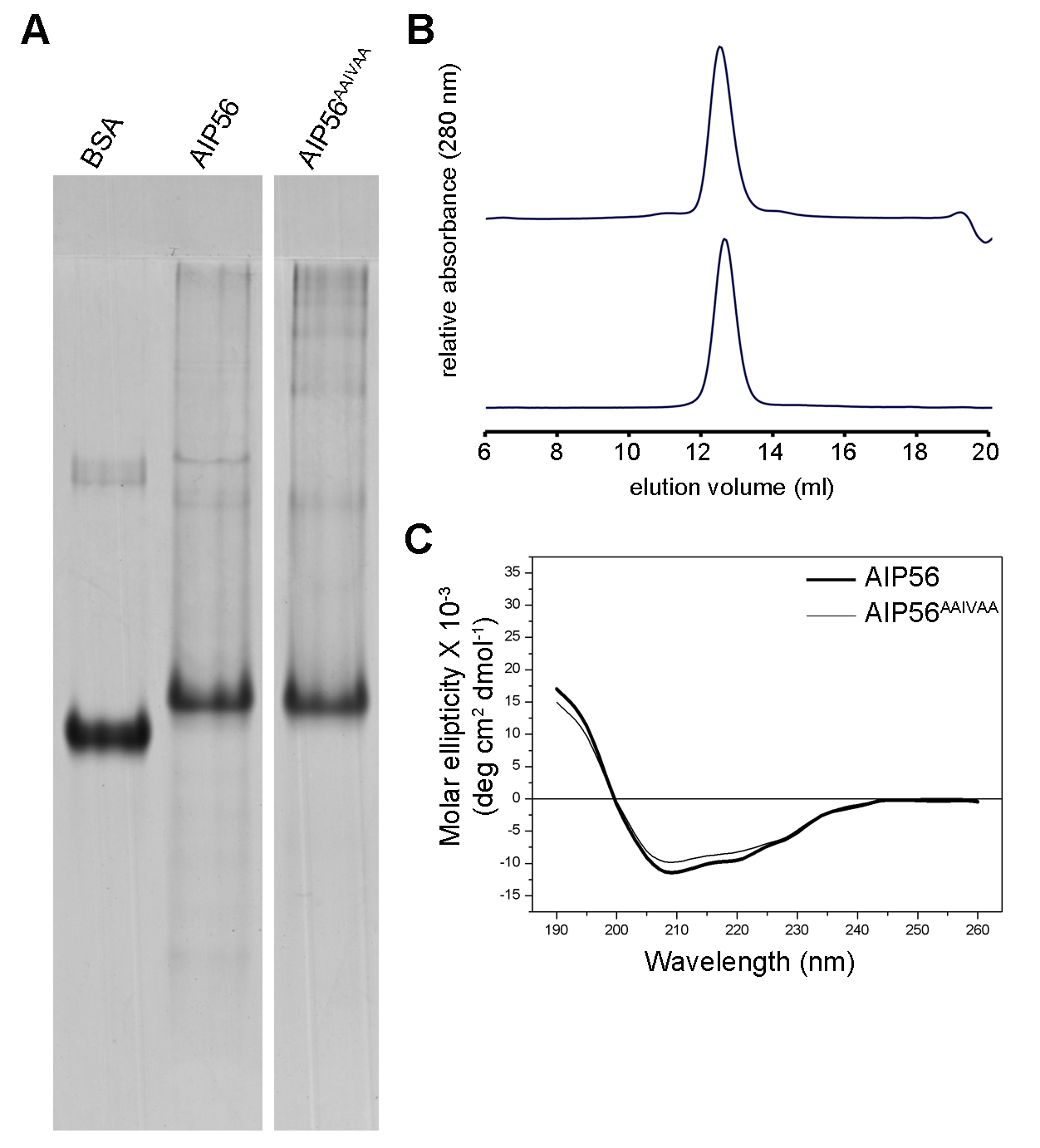

Supplement: Figure S2 — Disruption of the zinc-metalloprotease signature does not induce major structural changes in AIP56. Native-PAGE (A) and size exclusion chromatography (B) of AIP56 and AIP56AAIVAA showing that disruption of the zinc-binding motif did not affect the monodispersity/stokes radius of the protein. In Native-PAGE, BSA electrophoretic mobility is shown for reference purposes. (C) Far-UV CD spectra of wild-type AIP56 (thick line) and AIP56AAIVAA (thin line) showing that the secondary structure content of the toxin was also unaffected by the introduced mutations. (TIF) [file ppat.1003128.s002.tif]

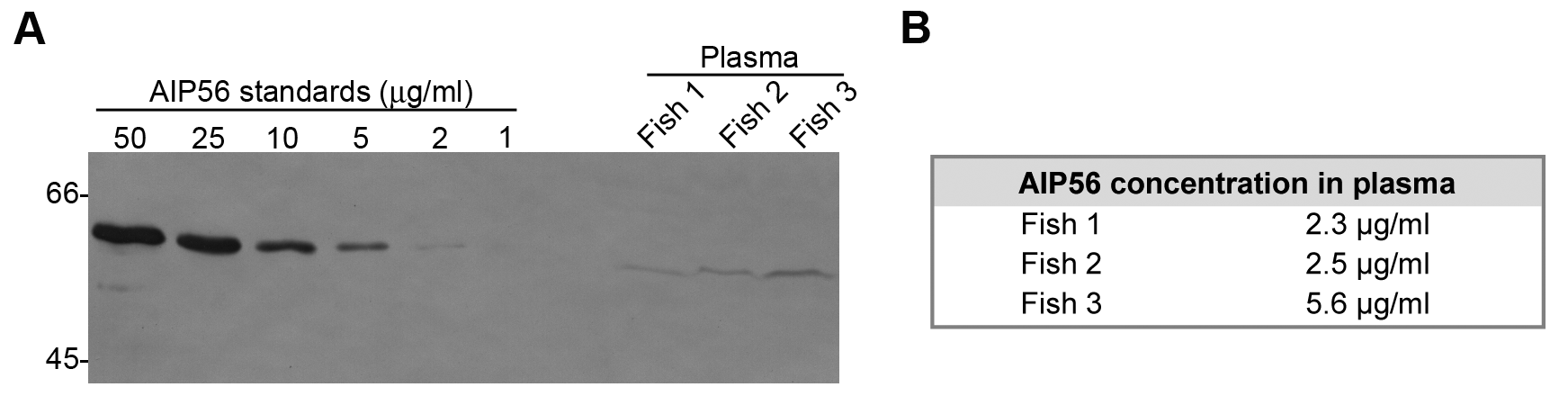

Supplement: Figure S3 — The AIP56 concentration in the plasma of infected fish is in the same range as those used in the present work. (A) The presence of AIP56 in plasmas (5 µl aliquots) from sea bass infected with a lethal dose of Phdp strain PP3 was determined by Western blotting. Different concentrations of recombinant AIP56 (5 µl) were loaded as standards. Numbers at the left refer to the position and mass (in kDa) of the molecular weight markers. (B) Concentrations of AIP56 in the plasmas analysed in (A), determined by densitometry, using a recombinant AIP56 standard curve. (TIF) [file ppat.1003128.s003.tif]

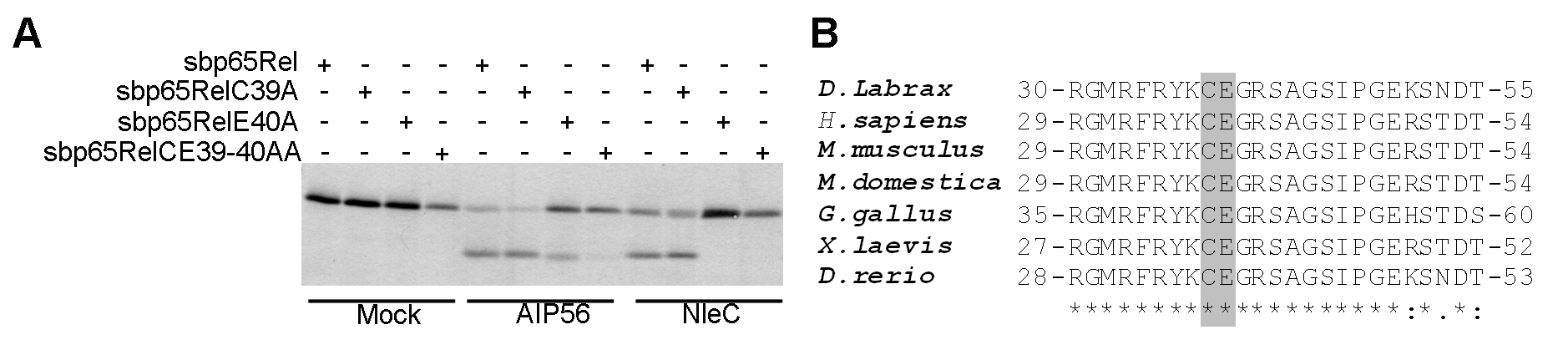

Supplement: Figure S4 — The AIP56 cleavage-determining residues are evolutionarily conserved. (A) Mutations in Cys39 and Glu40 of sbp65Rel inhibit proteolytic processing by AIP56 and NleC. 35S-labeled sbp65Rel, sbp65RelC39A, sbp65RelE40A or sbp65CE39-40AA were incubated for 2 h at 22°C with 100 nM of the indicated proteins and cleavage assessed by autoradiography. (B) Alignment of the p65 N-terminal region from different species (NCBI accession numbers: Homo sapiens, AAA36408; Mus musculus, NP_033071; Gallus gallus, NP_990460; Xenopus laevis, AAH70711; Monodelphis domestica, XP_001379658; Danio rerio, AAO26404). The residues mutated in (A) are shadowed grey. (TIF) [file ppat.1003128.s004.tif]

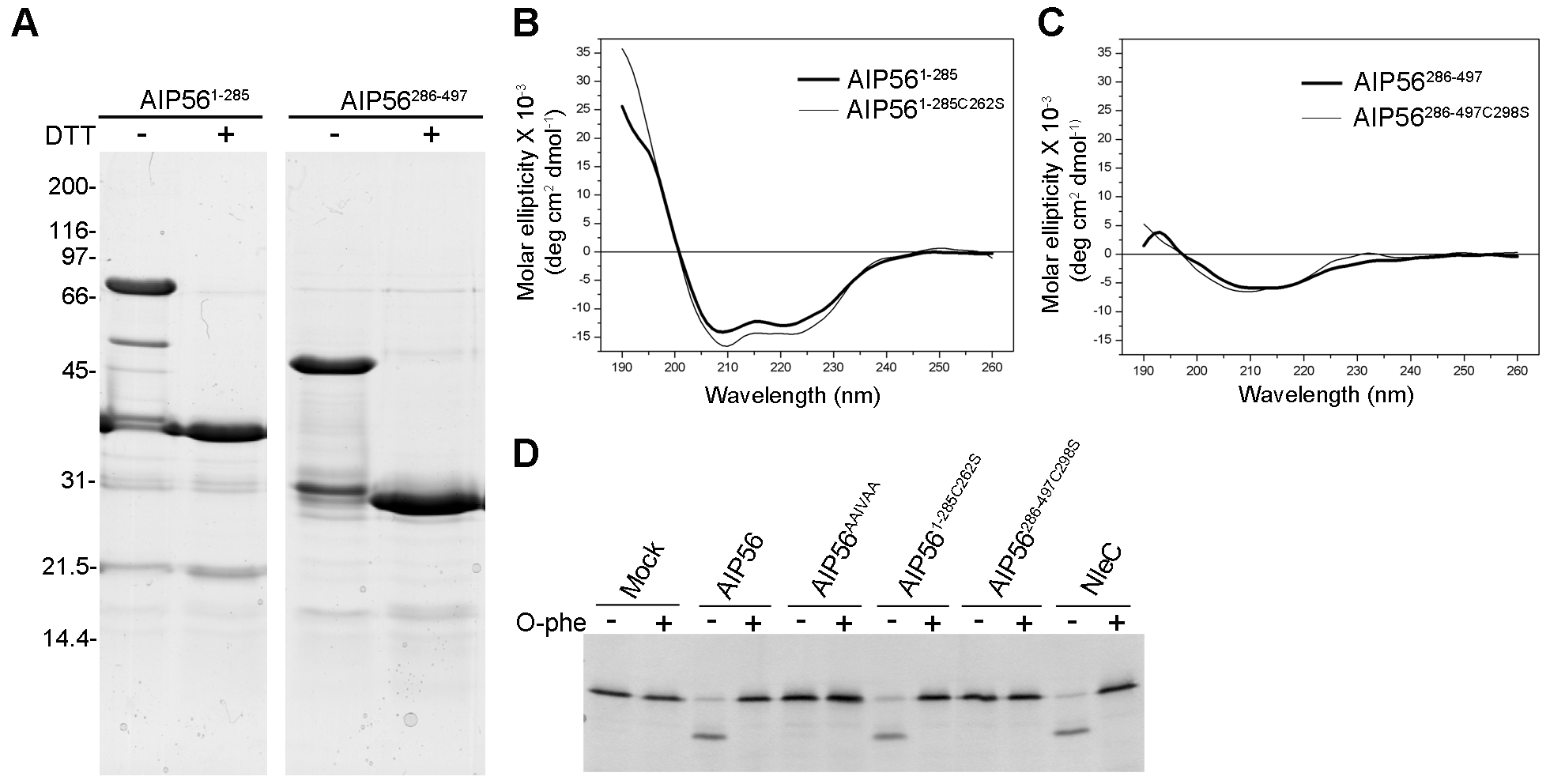

Supplement: Figure S5 — Structural and functional analysis of recombinant AIP56 N- and C-terminal domains. (A) Reducing and non-reducing SDS-PAGE of purified AIP561–285 and AIP56286–497. Numbers at the left refer to the position and mass (in kDa) of the molecular weight markers. (B) Far-UV CD spectra of AIP561–285 (thick solid line) and AIP561–285C262S (thin solid line). (C) Far-UV CD spectra of AIP56286–497 (thick solid line) and AIP56286–497C298S (thin solid line). (D) AIP56, AIP561–285C262S and NleC cleave sea bass p65 Rel homology domain in vitro. 35S-labeled sbp65Rel (Met1-Arg188) was incubated for 2 h at 22°C with 100 nM of the indicated proteins in the presence or absence of the metalloprotease inhibitor 1,10-phenanthroline (O-phe) and cleavage assessed by autoradiography. (TIF) [file ppat.1003128.s005.tif]

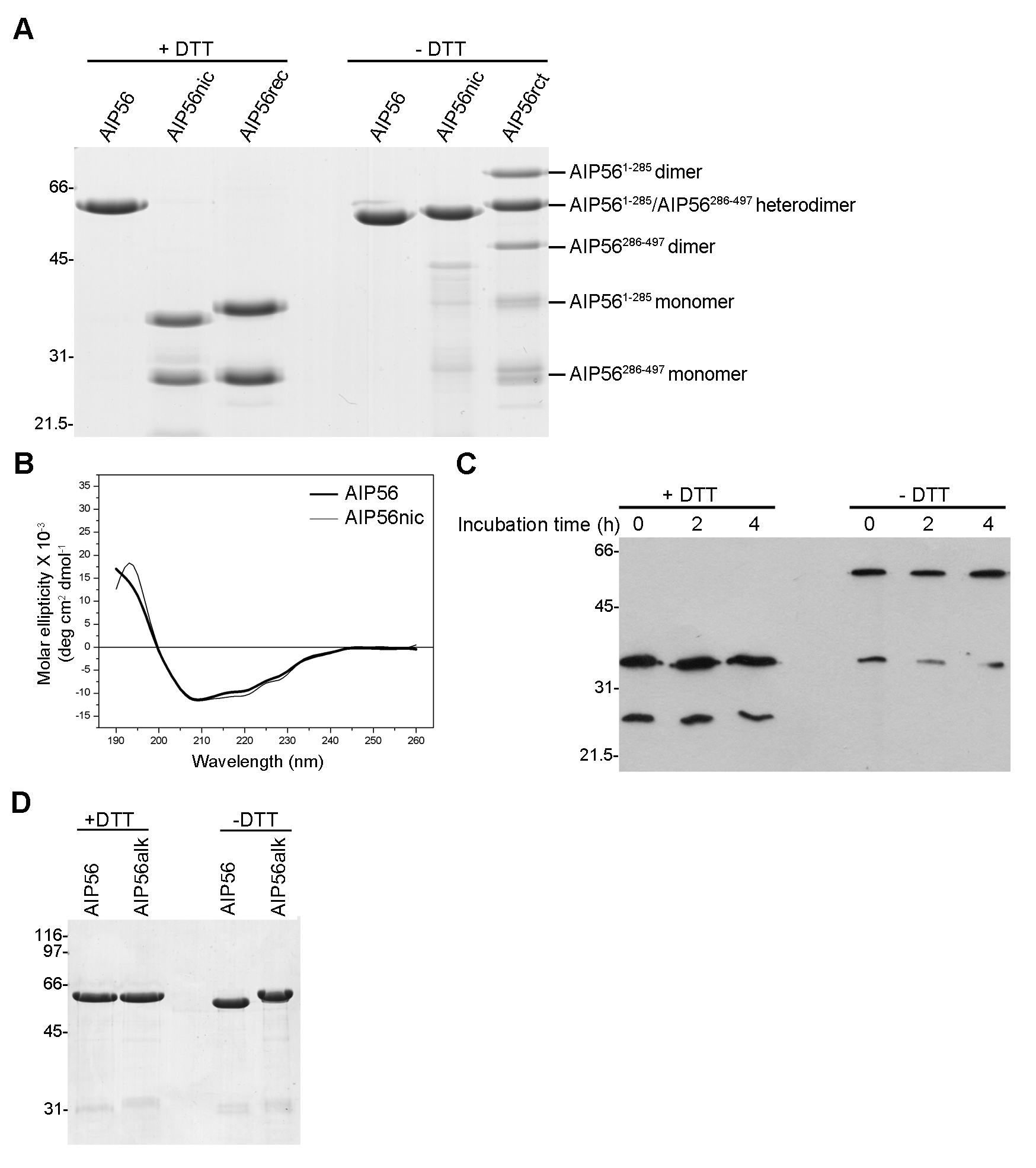

Supplement: Figure S6 — Analysis of nicked, reconstituted and alkylated AIP56. (A) Reducing (+DTT) and non-reducing (−DTT) SDS-PAGE of AIP56, nicked AIP56 (AIP56nic) and reconstituted AIP56 (AIP56rct). (B) Nicking of AIP56 does not affect its secondary structure. Far-UV CD spectra of AIP56 (thick line) and nicked AIP56 (AIP56nic; thin line). (C) The culture conditions do not reduce the disulphide bridge of nicked AIP56. Nicked AIP56 was added to a sea bass peritoneal cell suspension in supplemented L-15 medium and incubated up to 4 h at 22°C. An aliquot of nicked toxin not incubated with cells (0) and aliquots of the cell culture supernatant collected 2 or 4 h after incubation with cells (all containing 50 ng of nicked toxin) were run in reducing and non-reducing SDS-PAGE and subjected to Western blotting using an anti-AIP56 rabbit serum. (D) Reducing (+DTT) and non-reducing (−DTT) SDS-PAGE of AIP56 and alkylated AIP56 (AIP56alk). Numbers on the left of the panels indicate the mass of the molecular weight markers, in kDa. (TIF) [file ppat.1003128.s006.tif]
