# Supplementary material for: The Apoptogenic Toxin AIP56 Is a Metalloprotease A-B Toxin that Cleaves NF-κb P65
Source: PLoS Pathog. 2013 Feb 28;9(2):e1003128. doi: 10.1371/journal.ppat.1003128 (PMC3585134; doi:10.1371/journal.ppat.1003128)
Supplement: Materials and Methods S1 — Additional details about the constructs used in this study, protein production and purification, protein quantification, PAGE and Western blotting, analysis of the Zinc content, analytical size exclusion chromatography, circular dichroism spectroscopy (CD), differential scanning fluorimetry (DSF), determination of AIP56 concentration in the plasma of infected fish, are provided as Supporting Materials and Methods. (DOCX) [file ppat.1003128.s007.docx]

**MATERIAL AND Methods S1**

**Constructs**

For bacterial protein expression, DNA coding sequences were cloned into NcoI/XhoI restriction sites of pET-28a(+) (Novagen) in frame with a C-terminal 6xHis-tag. Mutated versions of the proteins were obtained by site directed mutagenesis using QuickChange® Site-Directed Mutagenesis Kit (Stratagene).

*AIP56 mutant (*AIP56^AAIVAA)^*:* plasmid pETAIP56^AAIVAA^, coding for His-tagged mutated version of AIP56, was obtained using pETAIP56H^+^ [[1](#_ENREF_1)] as template. The mutations consist on the substitution by Ala of the key residues for zinc ion coordination and water molecule activation: His^165^, Glu^166^, His^169^ and His^170^.

*AIP56 domains:* AIP56 putative A (AIP56^1-285^) and B (AIP56^286-497^) domains were designed based on the chymotrypsin cleavage site (see below). pETAIP56H^+^ [[1](#_ENREF_1)] was subjected to PCR amplification using primers AIP56Fw4NcoI/AIP56Rv7XhoI (Table S1) for AIP56^1-285^, and AIP56Fw6NcoI/AIP56Rv5XhoI (Table S1) for AIP56^286-497^. PCR fragments were cloned into pET-28a(+), yielding pETAIP56^1-285^ and pETAIP56^286-497^. Mutated versions of AIP56^1-285^ and AIP56^286-497^ with Cys^262^ or Cys^298^ mutated to Ser (AIP56^1-285C262S^ and AIP56^286-497C298S^) were obtained using either pETAIP56^1-285^ or pETAIP56^286-497^ as template.

*NleC:* the NleC coding sequence was amplified by PCR with primers EHECFw1NcoI and EHECRv1XhoI (Table S1) using total DNA [[2](#_ENREF_2)] from *E. coli* O157:H7 strain 4462 as template. PCR products were cloned into pET-28a(+).

*LF∙AIP56 chimeric proteins:* Chimeric proteins consist on the amino-terminus of anthrax lethal factor (LF^11-263^) fused to AIP56 N-terminal (LF^11-263^∙AIP56^1-261^) or AIP56 C-terminal (LF^11-263^∙AIP56^299-497^). The sequence encoding LF^11-263^ was amplified with primers LFFw1NcoI/LFRv1SacI (Table S1) from plasmid pRSET A (Invitrogen) containing LF gene [[3](#_ENREF_3)] and regions encoding AIP56^1-261^ and AIP56^299-497^ were amplified from pETAIP56H+ [[1](#_ENREF_1)] using the primer combinations NtermAIP56Fw1SacI**/**AIP56Rv9XhoI and CtermAIP56Fw1SacI/AIP56Rv5XhoI (Table S1), respectively. PCR fragments were digested with SacI, and LF^11-263^ ligated either with AIP56^1-261^ or AIP56^299-497^_._ Ligations were subjected to PCR using the primer combination LFFw1NcoI/AIP56Rv9XhoI or LFFw1NcoI/AIP56Rv5XhoI (Table S1) and PCR products were cloned into pET-28a(+).

sbp65Rel: The coding region (amino acids 1-188) of sea bass p65 REL domain (sbp65Rel) was amplified by PCR from cDNA produced as described in [[4](#_ENREF_4)] using DLp65Fw1NcoI with either DLp65Rv4XhoI or DLp65Rv2XhoI primers (table S1), for cloning His-tagged or untagged sbp65Rel, respectively. PCR products were cloned in pET-28a(+). The vector carrying his-tagged sbp65Rel was used for bacterial expression and the untagged version for production of ^35^S-labeled sbp65Rel.

**Protein production and purification**

Recombinant His-tagged proteins were expressed in *E. coli* BL21(DE3) cells grown in Lysogenic broth (LB) [[5](#_ENREF_5)] supplemented with 50 μg/ml kanamycin. Protein expression was induced with 1 mM Isopropyl β-D-1-thiogalactopyranoside (IPTG) and carried out overnight at 17 ºC, except for AIP56^AAIVAA^, AIP56^1-285C262S^ and sbp65Rel, which were expressed at 37 ºC for 4 h. Starting cultures were grown overnight at 37 ºC, used to inoculate fresh medium at 1:100 dilution and grown at 37 ºC. For expressions at 17 ºC, cultures were equilibrated to this temperature prior to induction. Protein expression was induced when bacterial cultures reached an OD of approximately 0.6. Induced bacterial cells were collected by centrifugation, resuspended in 50 mM phosphate buffer pH 7.4, 500 mM NaCl, 200 µg/ml lysozyme, 250 µg/ml PMSF and lysed by freeze/thaw followed by sonication in the presence of 10 μg/ml DNase I, and 10 mM MgCl_2_.

Affinity chromatography was performed in HisTrap HP columns (GE Healthcare) or using HIS-Select® Nickel Affinity Gel (Sigma) for sbp65Rel purification. Anion exchange chromatography was performed in a Bio-Scale^TM^ Unosphere Q cartridge (BioRad) and size exclusion chromatography on Sephacryl S100 HR column (GE Healthcare) except for AIP56^AAIVAA^ that was purified in a Sephacryl S200 HR column (GE Healthcare). Purified proteins were concentrated (5-10 mg/ml) using Amicon Ultra-15 Centrifugal Filter Units, frozen in liquid nitrogen and stored at -80 ºC. Protein purity was evaluated by SDS-PAGE followed by Coomassie-blue staining.

*AIP56:* production of soluble recombinant His-tagged AIP56 was adapted from [[1](#_ENREF_1)]. AIP56 was purified from the soluble fraction by affinity chromatography. Elution was carried out with increasing concentrations of imidazole in 50 mM phosphate buffer pH 7.4, 500 mM NaCl. Fractions containing AIP56 were pooled and applied to an anion exchange chromatography using a linear NaCl gradient (50 mM to 1 M) in 20 mM Tris-HCl pH 8.0. Fractions containing AIP56 were pooled, concentrated and stored in 10 mM Tris-HCl pH 8.0, 200 mM NaCl.

*AIP56 mutant (*AIP56^AAIVAA)^*:* AIP56^AAIVAA^ was purified from inclusion bodies, solubilized in 8 M urea, 20 mM Tris-HCl pH 8.0, 500 mM NaCl, 1 mM 2-mercaptoethanol, 5 mM imidazole and purified by affinity chromatography under denaturing conditions. The protein was eluted in the above buffer with increasing concentrations of imidazole. Fractions containing AIP56^AAIVAA^ were pooled, adjusted to 0.1 mg/ml, and subjected to refolding through dialysis against 3 x 50 volumes of sea bass PBS (sbPBS; phosphate buffer saline with osmotic strength adjusted to 322 mOsm) with 10 % glycerol, at 4 ºC. The refolded protein was purified by size exclusion chromatography in 20 mM Tris-HCl pH 8.0, 200 mM NaCl, 10 % glycerol. Fractions containing AIP56^AAIVAA^ were pooled, concentrated and stored in 20 mM Tris-HCl pH 8.0, 200 mM NaCl, 10 % glycerol.

*AIP56 truncates:* Production and purification of AIP56^1-285^ and AIP56^286-497^ were done as described for AIP56. AIP56^1-285C262S^ was purified from inclusion bodies as described for AIP56^AAIVAA^. AIP56^286-497C298S^ was purified from the soluble fraction by affinity chromatography as described for AIP56 but adding 10 % glycerol to elution buffers. Fractions containing AIP56^286-497C298S^ were pooled and purified by size exclusion chromatography as described for AIP56^AAIVAA^. AIP56^1-285^ and AIP56^286-497^ were stored in 10 mM Tris-HCl pH 8.0, 200 mM NaCl while AIP56^1-285C262S^ and AIP56^286-497C298S^ were stored in 20 mM Tris-HCl pH 8.0, 200 mM NaCl, 10 % glycerol.

*NleC:* NleC was purified from the soluble fraction by affinity chromatography as described for AIP56 but using 20 mM phosphate buffer pH 8.0, 500 mM NaCl with increasing concentrations of imidazole for protein elution. Fractions containing NleC were pooled and subjected to size exclusion chromatography in 20 mM Tris-HCl pH 8.0, 50 mM NaCl. Fractions containing NleC were pooled, concentrated and stored in 20 mM Tris-HCl pH 8.0, 50 mM NaCl.

*LF∙AIP56 chimeric proteins:* Chimeric LF^11-263^∙AIP56^1-261^ and LF^11-263^∙ AIP56^299-497^ were purified by affinity chromatography as described for AIP56. In the case of LF^11-263^∙AIP56^1-261^, to avoid the formation of protein aggregates, sonication of cell lysates was avoided and glycerol (5% final concentration) was added to cell lysates and affinity chromatography buffers. The chimeras were concentrated and stored in 10 mM Tris pH 8.0, 200 mM NaCl.

Sbp65Rel: His-tagged sbp65Rel was purified from the soluble fraction of induced bacterial cells by affinity chromatography through elution with increasing concentrations of imidazole in 50 mM phosphate buffer pH 7.4, 300 mM NaCl. Fractions containing sbp65Rel were pooled, concentrated and stored in 50 mM phosphate buffer pH 7.4, 300 mM NaCl.

**Protein quantification**

Reconstituted heterodimeric protein (AIP56^1-285^ linked to AIP56^286-497^ by a disulfide bridge) was quantified by densitometry analysis of coomassie-blue stained gels using BSA standards. The other proteins were quantified by measuring absorbance at 280 nm and using the extinction coefficient calculated by the ProtParam tool (http://www.expasy.org/tools/protparam.html), using the Edelhoch method [[6](#_ENREF_6)], but with the extinction coefficients for Trp and Tyr determined by Pace et al [[7](#_ENREF_7)].

**PAGE and Western blotting**

SDS- and Native-PAGE were performed using the Laemmli discontinuous buffer system [[8](#_ENREF_8)] as described in [[1](#_ENREF_1)]. Proteins were transferred onto nitrocellulose membranes and probed with anti-AIP56 [[1](#_ENREF_1)] or anti-sea bass NF-κB p65 rabbit sera (produced using the peptide SIFNSGNPARFVS located at the C-terminal region of sea bass p65 as antigen). Reactive bands were detected using anti-rabbit IgG alkaline phosphatase conjugate (Sigma) followed by BCIP/NBT development or using an anti-rabbit IgG horseradish peroxidase-linked secondary antibody (The binding site) followed by detection with SuperSignal^®^ West Dura Extended Duration Substrate (Pierce biotechnology). Blots shown are representative of at least three independent experiments.

**Analysis of Zinc content**

The zinc content was determined by atomic absorption spectroscopy with flame optimization in an Atomic Absorption Spectrometer PU 9200X (Philips). All buffers were prepared with chemicals of the highest purity available and using Milli-Q grade water. All material was previously immersed for 24 hours in 10% nitric acid, washed with Milli-Q grade water and autoclaved in paper bags. Before metal determination, protein samples (0.1-0.2 mg/ml) were extensively dialyzed against 10 mM Tris-HCl pH 8.0, 200 mM NaCl using Amicon Ultra-15 centrifugal filter devices (Millipore). Metal content was measured after standardization in the linear concentration range (0-1 ppm). Results are expressed as the mean±SD of three independent measurements carried out in triplicate.

**Analytical size exclusion chromatography**

Protein samples in 10 mM Tris-HCl pH 8.0, 200 mM NaCl were subjected to analytical size exclusion chromatography in a Superose 12 10/300 column (GE Healthcare) using an AKTA Purifier FPLC system (Pharmacia) at room temperature and a 0.5 ml/minute flow rate. The column was pre-equilibrated in the above buffer and protein elution was monitored by measuring the absorbance at 280 nm. Molecular weights of eluted proteins were estimated based on column calibration with molecular weight/stokes radius standards.

**Circular dichroism Spectroscopy (CD)**

Far UV CD spectra were acquired on an Olis DSM 20 circular dichroism spectropolarimeter continuously purged with nitrogen, equipped with a Quantum Northwest CD 150 Temperature-Controlled cuvette and controlled by the Globalworks software. Scans were collected at 20 °C with a 0.2 mm path length cuvette between 190 and 260 nm at 1 nm intervals. Three scans with an integration time of 4 seconds were averaged for each measurement. Protein concentration was determined by absorbance measurements. Proteins were dissolved in 10 mM Tris-HCl pH 8.0, 50 mM NaCl. The results are expressed in terms of mean residue molar ellipticity [Θ]_MRW_ in deg cm^2^ dmol^-1^, according to the equation [Θ]_MRW_ = Θ_obs_ * MW * 100 / (l * c * N) where Θ_obs_ is the observed ellipticity in deg, MW is the protein molecular weight in g/mol, l is the cuvette path length in cm, c is the protein concentration in g/l and N is the number of residues of the protein. Analysis of the protein secondary structure was performed using the Globalworks software algorithm.

**Differential scanning fluorimetry (DSF)**

The unfolding of AIP56 and nicked AIP56 was monitored by following SYPRO® Orange (Invitrogen) fluorescence. Proteins samples in phosphate/citrate buffer, 150 mM NaCl pH 7.0 were mixed 1:1 (v/v) with dye solution in the same buffer in a final volume of 30 μl and analyzed in white 96-well plates on a iQ5 Real Time PCR System (BioRad) by measuring fluorescence at 585 nm as a function of temperature (scanned from 20 to 95 ºC in 0.5 ºC/min steps). AIP56 and nicked AIP56 were used at 5 μM with 10 x SYPRO® Orange. Controls included no protein and/or no dye. The melting curves were analyzed using CFX Manager (BioRad) and the melting temperature (T_m_) was calculated as the inflection point of the curve of 16 measurements in 4 independent experiments.

**Determination of the AIP56 concentrations in the plasma of infected fish**

Sea bass weighting 16.3±2.4 g were infected i.p. with a lethal dose (1.8x10^6^ CFU/fish) of *Phdp* strain PP3 [[9](#_ENREF_9)]. Growth conditions and inoculum preparation were carried out as described [[1](#_ENREF_1),[10](#_ENREF_10)]. Plating serial dilutions of the final bacterial suspensions onto TSA-1 plates and counting the number of CFU following incubation at 22ºC for 2 days confirmed bacterial concentrations of the inoculum. Plasmas were collected from moribund fish as previously described [[9](#_ENREF_9)]. Five microliters aliquots of the plasmas or recombinant AIP56 standards (at 50, 25, 10, 2 and 1 µg/ml) were analyzed by Western blotting. The concentrations of AIP56 in the plasmas were determined by densitometry using a recombinant AIP56 standard curve.

REFERENCES

1. do Vale A, Silva MT, dos Santos NM, Nascimento DS, Reis-Rodrigues P, et al. (2005) AIP56, a novel plasmid-encoded virulence factor of *Photobacterium damselae* subsp. *piscicida* with apoptogenic activity against sea bass macrophages and neutrophils. Mol Microbiol 58: 1025-1038.

2. Sambrook J, Russel DW (2001) Molecular cloning: a laboratory manual. New York: Cold Spring Harbor Laboratory Press.

3. Zornetta I, Brandi L, Janowiak B, Dal Molin F, Tonello F, et al. (2010) Imaging the cell entry of the anthrax oedema and lethal toxins with fluorescent protein chimeras. Cell Microbiol 12: 1435-1445.

4. Pinto RD, da Silva DV, Pereira PJ, dos Santos NM (2012) Molecular cloning and characterization of sea bass (Dicentrarchus labrax, L.) Tapasin. Fish Shellfish Immunol 32: 110-120.

5. Bertani G (2004) Lysogeny at mid-twentieth century: P1, P2, and other experimental systems. J Bacteriol 186: 595-600.

6. Edelhoch H (1967) Spectroscopic determination of tryptophan and tyrosine in proteins. Biochemistry 6: 1948-1954.

7. Pace CN, Vajdos F, Fee L, Grimsley G, Gray T (1995) How to measure and predict the molar absorption coefficient of a protein. Protein Sci 4: 2411-2423.

8. Laemmli UK (1970) Cleavage of structural proteins during the assembly of the head of bacteriophage T4. Nature 227: 680-685.

9. do Vale A, Costa-Ramos C, Silva A, Silva DS, Gartner F, et al. (2007) Systemic macrophage and neutrophil destruction by secondary necrosis induced by a bacterial exotoxin in a Gram-negative septicaemia. Cell Microbiol 9: 988-1003.

10. do Vale A, Marques F, Silva MT (2003) Apoptosis of sea bass (*Dicentrarchus labrax* L.) neutrophils and macrophages induced by experimental infection with *Photobacterium damselae* subsp*. piscicida*. Fish Shellfish Immunol 15: 129-144.
