# Supplementary material for: The Apoptogenic Toxin AIP56 Is a Metalloprotease A-B Toxin that Cleaves NF-κb P65
Source: PLoS Pathog. 2013 Feb 28;9(2):e1003128. doi: 10.1371/journal.ppat.1003128 (PMC3585134; doi:10.1371/journal.ppat.1003128)
Supplement: Table S1 — Primers used in this study. (DOCX) [file ppat.1003128.s008.docx]

Table S1. Primers used in this study.

| **Primer designation** | **Nucleotide sequence 5′→3′** |
| --- | --- |
| **AIP56EHECFw1BstBI** | cgcTTCGAAAGGCTGGGTACGATCAGTGACCGATATGAGGCTTCGCCTGACTTCGGCACCCTGACCTCTTTT |
| **AIP56Fw4NcoI** | GCGCCATGGACAACGATAAACCAGATGCAAGC |
| **AIP56Fw6NcoI** | GCGCCATGGTTCTCCCTAGCGCTAGCGCCG |
| **AIP56Rv5XhoI** | GCGCTCGAGATTAATGAATTGTGGCGCGTGGG |
| **AIP56Rv7XhoI** | GAGCTCGAGAAAGGCGCCGCCCCCGTTG |
| **AIP56Rv9XhoI** | GAGCTCGAGAAAAGAGGTCAGGGTGCCGAA |
| **CtermAIP56Fw1SacI** | GCGGAGCTCACTTTTGATGTACTAAATCGAAT |
| **NtermAIP56Fw1SacI** | GCGGAGCTCAACAACGATAAACCAGATGCAAGC |
| **EHECFw1NcoI** | CATGCCATGGAAATTCCCTCATTACAG |
| **EHECRv1XhoI** | CCGCTCGAGTTGCTGATTGTGTTTGTC |
| **LFFw1NcoI** | GCGCCATGGTAAAAGAGAAAGAGAAAAATAAAG |
| **LFRv1SacI** | CGCGAGCTCCCGTTGATCTTTAAGTTCTTC |
| **DLp65Fw1NcoI** | CGCCCATGGAAGGTGTGTATGGATGGAGCCTG |
| **DLp65Rv4XhoI** | GCGCTCGAGCCTGTTGTCATAGATGGGCTGCG |
| **DLp65Rv2XhoI** | GCGCTCGAGTCACCTGTTGTCATAGATGGGCTGCG |
